# Effects of azo dye on simultaneous biological removal of azo dye and nutrients in wastewater

**DOI:** 10.1098/rsos.180795

**Published:** 2018-08-15

**Authors:** Aihui Chen, Bairen Yang, Yuanqiang Zhou, Yuze Sun, Cheng Ding

**Affiliations:** 1 School of Environmental Science and Engineering, Yancheng Institute of Technology, Yancheng, Jiangsu 224051, People's Republic of China; 2 Key Laboratory of Tideland Ecology and Pollution Control about Environmental Protection, Yancheng, Jiangsu 224051, People's Republic of China

**Keywords:** azo dye, decolorization, biological nitrogen removal, sequencing batch reactor

## Abstract

The potential disrupting effects of Azo dye on wastewater nutrients removal deserved more analysis. In this study, 15 days exposure experiments were conducted with alizarin yellow R (AYR) as a model dye to determine whether the dye caused adverse effects on biological removal of both the dye and nutrients in acclimated anaerobic–aerobic–anoxic sequencing batch reactors. The results showed that the AYR removal efficiency was, respectively, 85.7% and 66.8% at AYR concentrations of 50 and 200 mg l^−1^, while higher AYR inlet (400 mg l^−1^) might inactivate sludge. Lower removal of AYR at 200 mg l^−1^ of AYR was due to the insufficient support of electron donors in the anaerobic process. However, the decolorized by-products *p*-phenylenediamine and 5-aminosalicylic were completely decomposed in the following aerobic stage at both 50 and 200 mg l^−1^ of AYR concentrations. Compared with the absence of AYR, the presence of 200 mg l^−1^ of AYR decreased the total nitrogen removal efficiency from 82.4 to 41.1%, and chemical oxygen demand (COD) removal efficiency initially decreased to 68.1% and then returned to around 83.4% in the long-term exposure time. It was also found that the inhibition of AYR, nitrogen and COD removal induced by a higher concentration of AYR was due to the increased intracellular reactive oxygen species production, which caused the rise of oxidation–reduction potential value and decreased ammonia monooxygenase and nitrite oxidoreductase activities.

## 1. Introduction

Azo dyes are primary contaminants in dyeing process effluent (DE) [1,2]. Owing to their harmful health effects and environmental consequence, how to effectively remove azo dyes becomes a research focus in the field of water pollution control. Azo dyes are recalcitrant under aerobic conditions, but could be transformed into their corresponding aromatic amines under anaerobic conditions [3–5]. Haug *et al*. [4] suggested that an aerobic process should follow the anaerobic reduction to further degrade the poisonous aromatic amine by-products into simple compounds. Therefore, the anaerobic–aerobic sequential treatment process is a feasible approach towards achieving complete degradation of azo dyes [6].

Decolorization of azo dyes is an anaerobic reduction process involving four electrons transfer to break one azo bond. Previous studies have shown that the electron donors in the DE are insufficient and cannot meet the need of azo bond cleavage [7]. To improve the decolorization efficiency, additional electron donors, such as glucose, acetate, yeast extract, etc., are supplied during the anaerobic reduction process [8,9]. However, these substances are external supplements, which cause an extra operation cost.

In China, most of the wastewater treatment plants (WWTPs) are running with activated sludge anaerobic/aerobic sequential treatment processes, which can satisfy the procedure of DE treating [10]. Considering the potential of using domestic wastewater (DW) as an electron donor source [11], co-treating of DW with DE may not only enhance the removal efficiency of the azo dyes and other pollutants, but reduce the operation cost simultaneously. However, azo dyes exhibit physiological toxicity [9,12], so it is of great significance to find out the potential adverse effects of azo dyes on other nutrients removal and the sludge activity.

In this study, alizarin yellow R (AYR) was used as a model of the azo dyes. The effects of AYR on simultaneous biological removal of AYR and other nutrients were investigated in the acclimated anaerobic–aerobic–anoxic sequencing batch reactors (SBRs). The intracellular reactive oxygen species (ROS) production was measured to examine the oxidative stress in the presence of AYR. Scanning electron microscope (SEM) and lactate dehydrogenase (LDH) assays were used to evaluate the surface integrity of activated sludge. The oxidation–reduction potential (ORP) values were measured to determine the variation of reductive capability during the anaerobic process. The activities of key enzymes involved in biological nitrogen removal were measured to explore the potential influences of AYR on nitrification and denitrification processes. Finally, the mechanisms of AYR depressing AYR and other nutrients removal were determined.

## 2. Material and methods

### 2.1. Parent sequencing batch reactors operation

Four parent SBRs (SBR1, SBR2, SBR3 and SBR4), with a working volume of 4.0 l, were seeded with biomass from Chengdong municipal WWTP in Yancheng, China. The SBRs were operated at 21 ± 2°C with three 8 h cycles per day. Each cycle consisted of 2.5 h anaerobic, 3 h aerobic and 1 h anoxic periods, followed by 1 h settling, 5 min decanting and 25 min idle periods. In the first 10 min of the anaerobic stage, each reactor was fed with 3 l of DW. In the aerobic time, the air was provided intermittently using an on/off control system with an online DO detector to keep the DO concentration in the reactor around 2.0 mg l^−1^. Before the start of settling, sludge was wasted to maintain the solids retention time of 22 days. After the settling period, 3 l of supernatant was discharged. The reactor was constantly mixed with magnetic stirrer except for the settling, decanting and idle periods.

### 2.2. Alizarin yellow R exposure experiments

After all the four parent SBRs had been operated over 100 days with DW and achieved stable biological nitrogen removal efficiency (greater than 80%), SBR1, SBR2 and SBR3 were fed with AYR-added DW, while SBR4 was operated as the control without the addition of AYR. The AYR added to the DW resulting in the AYR concentrations in the influent of SBR1, SBR2 and SBR3 were 50, 200 and 400 mg l^−1^, respectively. All other operational conditions were the same as the parent SBRs.

### 2.3. Diluting the domestic wastewater

The DW was collected from a sewage well of Yancheng Institute of Technology, China, and then was filtered by a 200-mesh sieve to remove particles. To simulate the real co-treating of DE with DW, tap water was added to make sure the initial concentration of chemical oxygen demand (COD) decreasing to be approximately at 400 mg l^−1^, and other key parameters of the diluted DW were listed in electronic supplementary material, table S1.

### 2.4. Analytic methods

Liquid samples taken from SBRs were immediately filtered through a 0.45 µm filter. AYR concentration was quantified by a UV–Vis spectrophotometer (Shimadzu UV-2550, Japan) at a wavelength of 375 nm [13]. *p*-Phenylenediamine (PPD) and 5-aminosalicylic (5-ASA) were measured by high-performance liquid chromatography (HPLC) equipped with a Waters Symmetry C18 column (150 × 4.6 mm, 5 µm) and a UV–Vis detector. The detailed procedures of PPD and 5-ASA were conducted according to the literature [14,15]. ORP value was detected with an ORP meter (Hanna HI8424, Italy) equipped with a redox electrode. The measurements of NH_4_^+^–N, NO_3_^−^–N, NO_2_^−^–N, total nitrogen (TN), ammonia monooxygenase (AMO), nitrite oxidoreductase (NOR), nitrate reductase (NAR) and nitrite reductase (NIR) activities were carried out according to the methods reported in previous publications [16,17].

SEM images of the activated sludge were obtained using the Hitachi 3400 SEM at 15 kV. The detailed procedure of pretreating the sludge is as follows. At the end of the 15 days exposure, aliquots were centrifuged at 100*g* for 5 min. The pellet was washed three times with 0.1 M phosphate buffer (pH 7.4) and then fixed 4 h in 0.1 M phosphate buffer (pH 7.4) containing 2.5% glutaraldehyde at 4°C. After rinsing twice with 0.1 M phosphate buffer (pH 7.4), the pellets were dehydrated in ethanol serials (50, 70, 80, 90 and 100%, 15 min per step), and then air-dried.

ROS production was determined using an established fluorescence assay [18]. LDH release was measured by the cytotoxicity detection kit (Roche Molecular Biochemicals) according to the manufacturer's instruction. The determinations of sludge volume index, mixed liquor suspended solids and mixed liquor volatile suspended solids were conducted in accordance with the standard methods [19].

### 2.5. Statistical analysis

All tests were performed in triplicate and the results were expressed as a mean ± standard deviation. An analysis of variance was used to test the significance of results, and *p* < 0.05 was considered to be statistically significant.

## 3. Results and discussion

### 3.1. Alizarin yellow R decolorization in simultaneous biological wastewater treatment system

**Figure 1.**
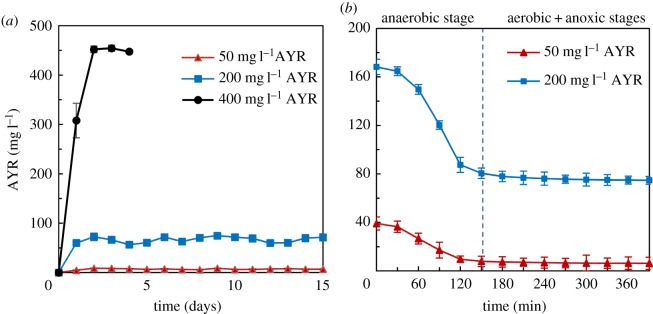
The variations of AYR in the 15 days culture (*a*) and during one cycle (*b*) in the acclimated activated sludge culture. Error bars represent standard deviations of triplicate tests.

Figure 1*a* presents the effluent AYR concentrations in the 15 days cultivation at AYR concentrations of 50, 200 and 400 mg l^−1^. When the concentration of inlet AYR was 50 mg l^−1^ (SBR1), the average effluent concentration of AYR was 6.3 ± 0.8 mg l^−1^, and the corresponding decolorization efficiency was about 85.7%. When AYR concentration was 200 mg l^−1^ (SBR2), the effluent concentration of AYR approximately increased to 74.7 ± 4.7 mg l^−1^, and the corresponding AYR decolorization efficiency of SBRS 2 was around 66.8%, which were significantly lower than that in the SBR1 (85.7%) (*p* < 0.05). However, when AYR concentration was up to 400 mg l^−1^ (SBR3), the effluent concentration of AYR increased to 447.4 ± 2.4 mg l^−1^ and there was no net AYR removal, also, it was found that the sludge was inactivated. Apparently, the decolorization efficiency of the SBRs was negatively related to the inlet concentration of AYR and more than 200 mg l^−1^ of inlet AYR was too much for the SBR reactor to handle. In the following section, only the AYR concentrations of 50 and 200 mg l^−1^ were considered.

Figure 1*b* presents the variation of effluent AYR during a treating cycle at AYR concentrations of 50 and 200 mg l^−1^. It can be seen from figure 1*b* that AYR concentration decreased significantly from 39.1 ± 1.54 to 7.9 ± 4.3 mg l^−1^ in SBR1 and from 168.2 ± 6.3 to 80.5 ± 4.3 mg l^−1^ in SBR2 at the anaerobic stage, and the corresponding decolorization efficiencies were 79.8 and 52.1%, respectively. Moreover, at the end of the anoxic stage, the concentrations of AYR in SBR1 and 2 were at 6.3 ± 5.0 and 74.1 ± 3.2 mg l^−1^, respectively. Only about 4.1 and 3.8% of AYR were further transferred at the aerobic and anoxic stages. These results indicated that the decolorization of AYR mainly happened at the anaerobic stage, which was in accordance with previous studies that the cleavage of azo bond in AYR is resistant to aerobic oxidation [3–5]. As the anaerobic stage is the key step for AYR decolorization, to improve the AYR decolorization efficiency, the anaerobic stage was prolonged to 3.5 h in SBR2. Nevertheless, compared with 2.5 h of the anaerobic stage, there was no significant promotion of the decolorization efficiency of AYR (*p* > 0.05) (electronic supplementary material, figure S1). The possible reasons were discussed in the next section.

### 3.2. The fate of alizarin yellow R decolorized products *p*-phenylenediamine and 5-aminosalicylic in the wastewater treatment system

**Figure 2.**
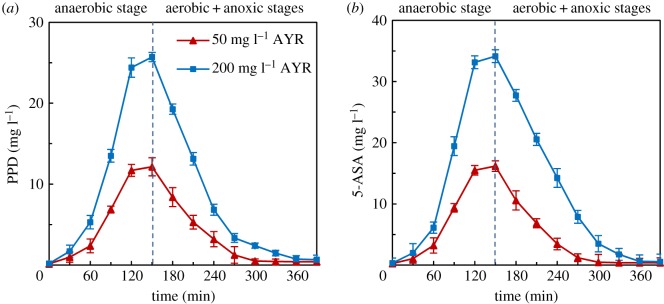
Effects of AYR on the variations of PPD (*a*) and 5-ASA (*b*) during one cycle in the acclimated activated sludge culture. Error bars represent standard deviations of triplicate tests.

PPD and 5-ASA are the two main products of AYR decolorization in the anaerobic reduction [14,15]. As can be seen in figure 2, in SBR2, at the end of the anaerobic stage, the PPD and 5-ASA contents increased to 25.67 ± 1.59 and 34.15 ± 1.10 mg l^−1^, respectively, which were much higher than those at SBR1 (12.14 ± 1.08 and 16.21 ± 0.89 mg l^−1^) (*p* < 0.05). In the subsequent aerobic stage, PPD and 5-ASA were quickly degraded. In both 50 and 200 mg l^−1^ inlet AYR concentrations, aerobic process exhibited excellent PPD and 5-ASA degradation. At the end of the anoxic stage, in SBR1 and 2, the residual PPD and 5-ASA decreased to 0.40 ± 0.35 and 0.37 ± 0.81 mg l^−1^, and 0.67 ± 0.56 and 0.54 ± 1.24 mg l^−1^, respectively, which were under the limit concentrations in the textile wastewater discharge standard GB 4287-2012 in China. As the great quantity of PPD and 5-ASA produced in SBR2 in the anaerobic stage were easy to be mineralized in the subsequent anaerobic process, thus the step of PPD and 5-ASA mineralization was not an important limiting step of the AYR mineralization. However, in SBR2, only about 52.1% of AYR was transferred to PPD and 5-ASA. Therefore, it was concluded that AYR decolorized to PPD and 5-ASA in the anaerobic stage is the key step of AYR degradation.

### 3.3. Effects of alizarin yellow R on chemical oxygen demand and nitrogen removal

**Figure 3.**
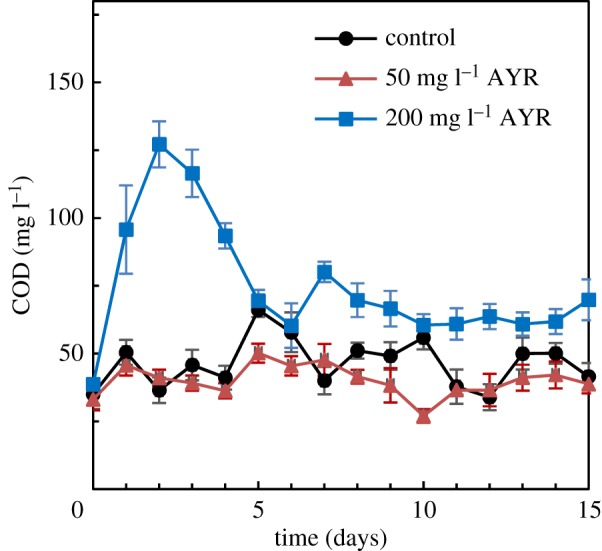
Effects of AYR on the variations of COD during the 15 days culture. Error bars represent standard deviations of triplicate tests.

As shown in figure 3, the effluent COD concentrations at AYR concentration of 50 mg l^−1^ (SBR1) were relatively stable, and the average effluent COD concentrations were 32.7 ± 3.3 mg l^−1^, and the corresponding COD removal efficiency was 89.9%, which was not different from the control SBR (88.2%) (*p* > 0.05), suggesting that 50 mg l^−1^ inlet AYR showed no measurable effect on COD removal. For SBR2 with 200 mg l^−1^ of inlet AYR, the COD removal efficiency decreased dramatically to 68.1% in the initial 2 days of exposure; however, with the increasing of culture time to 5 days, COD removal efficiency in the SBR2 gradually increased to 82.5%, which was not different from the control SBR (83.2%) (*p* > 0.05). Moreover, in the following 10 days exposure, the COD removal efficiency of the SBR2 was around 83.4%. Nevertheless, the COD removal was inhibited in the initial 4 days of 200 mg l^−1^ AYR added. It was clear that higher concentration of AYR (200 mg l^−1^) did not inhibit the COD removal in the long-term exposure time.

**Figure 4.**
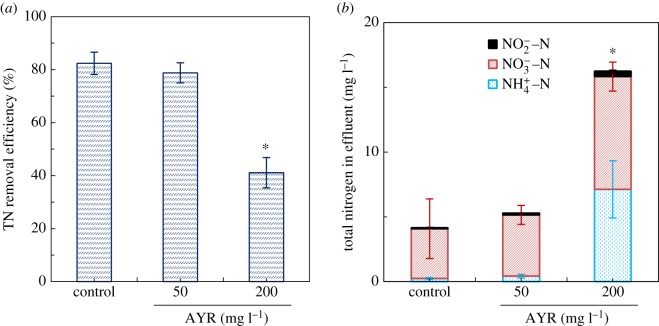
Effect of AYR on the total nitrogen (TN) removal (*a*) and the variation of NO_2_^−^–N, NO_3_^−^–N and NH_4_^+^–N in the effluent (*b*). Asterisks indicate statistical differences (*p* < 0.05) from the control test. Error bars represent standard deviations of triplicate tests.

The average TN removal efficiency was 78.8% at AYR concentration of 50 mg l^−1^, which was almost the same as that observed in the control SBR (82.4%), suggesting that 50 mg l^−1^ of AYR had no measurable effect on TN removal (figure 4*a*). However, when activated sludge was exposed to 200 mg l^−1^ of AYR, the effluent TN significantly increased to 22.2 ± 5.7 mg l^−1^, and the corresponding nitrogen removal efficiency was only about 41.1%, lower than that in the control SBR (82.4%) (*p* < 0.05). It seems that TN removal was inhibited by 200 mg l^−1^ of AYR.

It is well known that biological nitrogen removal depends on the successful nitrification and the subsequent denitrification [20]. As seen in figure 4*b*, when the concentration of AYR was 200 mg l^−1^, the effluent NH_4_^+^–N significantly increased from non-detectable to approximately 7.12 ± 2.21 mg l^−1^. The average removal efficiency of NH_4_^+^–N was 65.6%, significantly lower than that in the control SBR (greater than 98.3%) (*p* < 0.05). Clearly, 200 mg l^−1^ of AYR inhibited the process of nitrification. Also, the effluent NO_3_^−^–N highly increased from 3.85 ± 2.30 to 8.71 ± 1.12 mg l^−1^ (*p* < 0.05) (figure 4*b*). As shown in figure 2, in SBR2, at the end of the anaerobic stage, 25.67 ± 1.59 mg l^−1^ of PPD and 34.15 ± 1.10 mg l^−1^ of 5-ASA accumulated in the wastewater, but only 0.67 ± 0.56 mg l^−1^ of PPD and 0.54 ± 1.24 mg l^−1^ of 5-ASA was retained in the effluent. Thus, in SBR2, it can be seen that about 25.0 mg l^−1^ of PPD and 33.6 mg l^−1^ of 5-ASA were mineralized at the aerobic and anoxic stage. In theory, complete mineralization of 1 mg of PPD can produce 0.26 mg of NO_3_^−^–N, and 1 mg of 5-ASA can produce 0.09 mg of NO_3_^−^–N. The calculated NO_3_^−^–N production due to PPD and 5-ASA mineralization in the SBR2 was about 9.5 mg l^−1^. There was more 4.6 mg l^−1^ of NO_3_^−^–N mineralized in the SBR2 than in the control, which showed that the denitrification process was not depressed. The more accumulated NO_3_^−^–N apparently produced in the processes of PPD and 5-ASA aerobic oxidation. In summary, the increase in TN in effluent in SBR2 of 200 mg l^−1^ of AYR was not only due to the deterioration of ammonia oxidation, but to the accumulation of produced NO_3_^−^–N of PPD and 5-ASA in aerobic and anoxic stages.

### 3.4. Mechanisms of alizarin yellow R affecting alizarin yellow R and nitrogen removal

**Figure 5.**
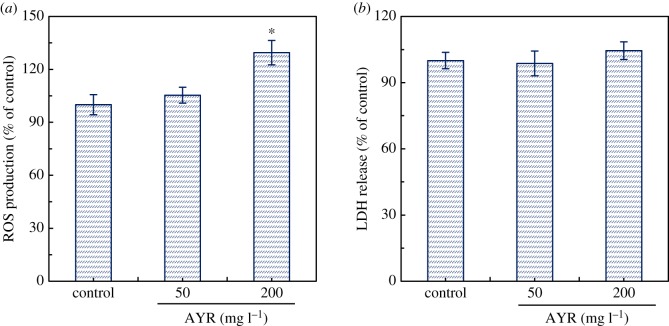
Effects of AYR on the intracellular ROS production (*a*) and LDH release (*b*). Asterisks indicate statistical differences (*p* < 0.05) from the control test. Error bars represent standard deviations of triplicate tests.

**Figure 6.**
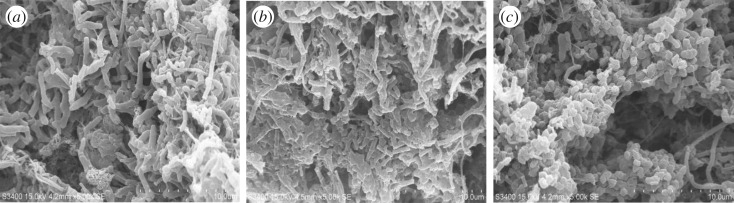
SEM images of activated sludge exposed to different concentrations of AYR. Control (*a*), 50 mg l^−1^ (*b*), 200 mg l^−1^ (*c*) of AYR.

It has been reported in the literature that azo dyes can cause oxidative stress and induce adverse effects on microbial community [9]. High ROS production may lead to the damage of cell membrane or cytoplasmic proteins in microbial cells [21]. In this study, compared with the control, the intracellular ROS production increased significantly at the AYR concentration of 200 mg l^−1^ (*p* < 0.05) (figure 5*a*). However, there was no obvious damage on the activated sludge surface (figure 6). The result of LDH release assay in figure 5*b* also confirmed that there was no measurable cell leakage at any concentrations of AYR (*p* > 0.05).

**Figure 7.**
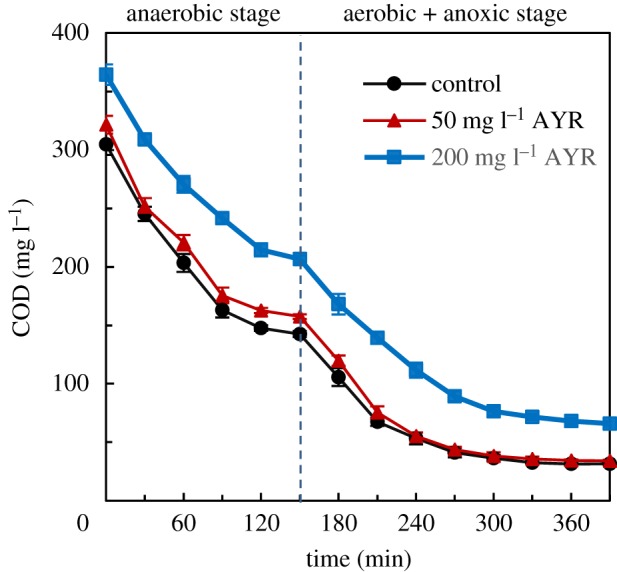
Effects of AYR on the variation of COD during one cycle in the acclimated activated sludge culture. Error bars represent standard deviations of triplicate tests.

Recent study has demonstrated the successful decomposition of azo dyes into aromatic amines due to sufficient electron donor source [22]. High ORP value inhibits the reduction process, and the redox potential values of azo dyes can be between −430 and −180 mV [23]. In this study, available COD in DW was the main electron donor source for both NO_3_^−^–N and AYR reduction. From figure 7, it can be seen that COD contents sharply decreased during the initial 2 h of the anaerobic process, and then there was no obvious decrease in COD concentration during the remaining anaerobic process at any concentrations of AYR. This result indicated that the capacity of DW to sufficiently provide electrons was reduced after 2 h of anaerobic culture. It was confirmed that the OPR value in all SBRs exceeded −180 mV after 2 h of anaerobic culture (electronic supplementary material, figure S2). These results explained the reason why prolonging the anaerobic time to 3.5 h was useless to the AYR removal.

The enzymes of AMO and NOR are responsible for the aerobic nitrification, whereas NAR and NIR are relevant to anaerobic denitrification [24–26]. As shown in table 1, there was no difference in the specific activities of NAR and NIR (*p* > 0.05) among all the three SBRs; but the presence of 200 mg l^−1^ of AYR decreased the activities of AMO and NOR (*p* < 0.05). These observations were in correspondence with higher effluent concentrations of NH_4_^+^–N at AYR concentration of 200 mg l^−1^ (figure 4). Also, as discussed above, NO_3_^−^–N was produced in the processes of PPD and 5-ASA aerobic oxidation, which may have resulted in the accumulation of NO_3_^−^–N in the effluent (figure 4). Therefore, the decreased biological nitrogen removal at higher concentration of AYR was not only attributed to the inhibition of AMO and NOR, but to the accumulated products of PPD and 5-ASA degradation.

**Table 1.** Activities of the key enzymes related to biological nitrogen removal in the presence of AYR^a^.

	NOR	AMO	NAR	NIR
control	0.082 ± 0.014	0.024 ± 0.003	0.048 ± 0.025	0.315 ± 0.043
50 mg l^−1^ AYR	0.079 ± 0.007	0.026 ± 0.002	0.047 ± 0.018	0.300 ± 0.050
200 mg l^−1^ AYR	0.028 ± 0.021	0.009 ± 0.003	0.041 ± 0.004	0.286 ± 0.035

^a^The unit is mol nitrite/(min mg protein). The data reported are the averages and their standard deviations of triplicate tests.

## 4. Conclusion

Co-treating of DE with DW was feasible, although the insufficient supply of electron donors in higher concentration (200 mg l^−1^) of AYR containing wastewater negatively affected the AYR decolorization. The intermediate by-products aromatic amines (PPD and 5-ASA) derived from AYR decolorization were further degraded in the following aerobic process. High concentration of AYR promoted intracellular ROS production, which caused the increase in OPR values and then inhibited the anaerobic reduction of AYR and other pollutants. Compared with the control, the presence of high concentration (200 mg l^−1^) of AYR depressed the biological nitrogen removal, which induced the accumulation of NH_4_^+^–N in the effluent. It was found that the increment of effluent NO_3_^−^–N was mainly derived from the decomposing of AYR. Further investigation revealed that the deterioration of ammonia oxidation was caused by the inhibited activities of nitrification-related enzymes of AMO and NOR.

## Supplementary Material

File contains calculation, Fig. S1, S2 and Table S1.;File for raw data

## Data accessibility

Further data can be obtained in the electronic supplementary material.

## References

[1] Brás R, Isabel MIA, Pinheiro HM, Goncalves IC 2001 Batch tests for assessing decolorisation of azo dyes by methanogenic and mixed cultures. J. Biotechnol. 89, 155–162. (10.1016/S0168-1656(01)00312-1) 11500209

[2] Cui MH, Cui D, Liang B, Sangeetha T, Wang AJ 2016 Decolorization enhancement by optimizing azo dye loading rate in an anaerobic reactor. RSC Adv. 6, 49 995–50 001. (10.1039/C6RA04665G)

[3] Weber EJ 1991 Studies of benzidine-based dyes in sediment-water systems. Environ. Toxicol. Chem. 10, 609–618. (10.1002/etc.5620100507)

[4] Haug W, Schmidt A, Nörtemann B, Hempel DC, Stolz A, Knackmuss HJ 1991 Mineralization of the sulfonated azo dye mordant yellow 3 by a 6-aminonaphthalene-2-sulfonate-degrading bacterial consortium. Appl. Environ. Microbiol. 57, 3144–3149. 1781678 10.1128/aem.57.11.3144-3149.1991 PMC183939

[5] Hu DX, Cui MH, Chen ZB, Tian Y, Cui YB, Ren NQ, Ran CQ, Sun HJ 2014 Performance of a novel HABR-CFASR system for the biological treatment of mixed printing and dyeing wastewater (MPDW). Desalin. Water Treat. 52, 5553–5562. (10.1080/19443994.2013.813005)

[6] Fernando E, Keshavarz T, Kyzaae G 2014 Complete degradation of the azo dye acid orange-7 and bioelectricity generation in an integrated microbial fuel cell, aerobic two-stage bioreactor system in continuous flow mode at ambient temperature. Bioresour. Technol. 156, 155–162. (10.1016/j.biortech.2014.01.036) 24495541

[7] Sani RK, Banerjee UC 1999 Decolorization of triphenylmethane dyes and textile and dyestuff effluent by *Kurthia* sp*.* Enzyme Microb. Technol. 24, 433–437. (10.1016/S0141-0229(98)00159-8)

[8] Khehra MS, Saini HS, Sharma DK, Chadha BS, Chimni SS 2005 Comparative studies on potential of consortium and constituent pure bacterial isolates to decolorize Azo dyes. Water Res. 39, 5135–5141. (10.1016/j.watres.2005.09.033) 16289280

[9] Rasool K, Mahmoud KA, Lee DS 2015 Influence of co-substrate on textile wastewater treatment and microbial community changes in the anaerobic biological sulfate reduction process. J. Hazard. Mater. 299, 453–461. (10.1016/j.jhazmat.2015.07.044) 26241771

[10] Chen ZB, Cui MH, Ren NQ, Chen ZQ, Wang HC, Nie SK 2011 Improving the simultaneous removal efficiency of COD and color in a combined HABMRCFASR system based MPDW. Part 1: optimization of operational parameters for HABMR by using response surface methodology. Bioresour. Technol. 102, 8839–8847. (10.1016/j.biortech.2011.06.089) 21778052

[11] Kim KY, Yang W, Logan BE 2015 Impact of electrode configurations on retention time and domestic wastewater treatment efficiency using microbial fuel cells. Water Res. 80, 41–46. (10.1016/j.watres.2015.05.021) 25996751

[12] Kolekar YM, Pawar SP, Gawai KR, Lokhande PD, Shouche YS, Kodam KM 2008 Decolorization and degradation of Disperse Blue 79 and Acid Orange 10, by *Bacillus fusiformis* KMK5 isolated from the textile dye contaminated soil. Bioresour. Technol. 99, 8999–9003. (10.1016/j.biortech.2008.04.073) 18562194

[13] Cui D, Guo YQ, Cheng HY, Liang B, Kong FY, Lee HS, Wang AJ 2012 Azo dye removal in a membrane-free up-flow biocatalyzed electrolysis reactor coupled with anaerobic bio-contact oxidation reactor. J. Hazard. Mater. 239–240, 257–264. (10.1016/j.jhazmat.2012.08.072) 23009797

[14] Cui D et al. 2014 Efficient azo dye removal in bioelectrochemical system and post-aerobic bioreactor: optimization and characterization. Chem. Eng. J. 243, 355–363. (10.1016/j.cej.2013.10.082)

[15] Cui MH, Cui D, Lee HS, Liang B, Wang AJ, Cheng HY 2016 Effect of electrode position on azo dye removal in an up-flow hybrid anaerobic digestion reactor with built-in bioelectrochemical system. Sci. Rep. 6, 25223 (10.1038/srep25223) 27121278 PMC4848485

[16] Zheng X, Wu R, Chen YG 2011 Effects of ZnO nanoparticles on wastewater biological nitrogen and phosphorus removal. Environ. Sci. Technol. 45, 2826–2832. (10.1021/es2000744) 21381661

[17] Zhu X, Chen YG 2011 Reduction of N**_2_**O and NO generation in anaerobic-aerobic (low dissolved oxygen) biological wastewater treatment process by using sludge alkaline fermentation liquid. Environ. Sci. Technol. 45, 2137–2143. (10.1021/es102900h) 21322643

[18] Limbach LK, Wick P, Manser P, Grass RN, Bruinink A, Stark WJ 2007 Exposure of engineered nanoparticles to human lung epithelial cells: influence of chemical composition and catalytic activity on oxidative stress. Environ. Sci. Technol. 41, 4158–4163. (10.1021/es062629t) 17612205

[19] APHA. 1998 Standard methods for the examination of water and wastewater, 20th edn Washington, DC: American Public Health Association.

[20] Chen A, Chen Y, Ding C, Liang H, Yang B 2005 Effects of tetracycline on simultaneous biological wastewater nitrogen and phosphorus removal. RSC Adv. 5, 59 326–59 334. (10.1039/c5ra08434b)

[21] Patra P, Roy S, Sarkar S, Mitra S, Pradhan S, Debnath N, Goswami A 2015 Damage of lipopolysaccharides in outer cell membrane and production of ROS-mediated stress within bacteria makes nano zinc oxide a bactericidal agent. Appl. Nanosic. 5, 857–886. (10.1007/s13204-014-0389-z)

[22] Dos Santos AB, Cervantes FJ, Van Lier JB 2007 Review paper on current technologies for decolorisation of textile wastewaters: perspectives for anaerobic biotechnology. Bioresour. Technol. 98, 2369–2385. (10.1016/j.biortech.2006.11.013) 17204423

[23] Dubin P, Wright KL 1975 Reduction of azo food dyes in cultures of *Proteus vulgaris*. Xenobiotica. 5, 563–571. (10.3109/00498257509056126) 1103488

[24] Juliette LY, Hyman MR, Arp DJ 1993 Inhibition of ammonia oxidation in *Nitrosomonas europaea* by sulfur compounds: thioethers are oxidized to sulfoxides by ammonia monooxygenase. Appl. Environ. Microbiol. 59, 3718–3727. 16349086 10.1128/aem.59.11.3718-3727.1993 PMC182523

[25] Kristjansson JK, Hollocher TC 1980 First practical assay for soluble nitrous oxide reductase of denitrifying bacteria and a partial kinetic characterization. J. Biol. Chem. 255, 704–707. 7356639

[26] Zumft WG 1997 Cell biology and molecular basis of denitrification. Microbiol. Mol. Biol. R. 61, 533–616. 10.1128/mmbr.61.4.533-616.1997 PMC232623 9409151

