# Supplementary material for: Effects of azo dye on simultaneous biological removal of azo dye and nutrients in wastewater
Source: R Soc Open Sci. 2018 Aug 15;5(8):180795. doi: 10.1098/rsos.180795 (PMC6124032; doi:10.1098/rsos.180795)
Supplement: File contains calculation, Fig. S1, S2 and Table S1.;File for raw data [file rsos180795supp1.docx]

**Supporting Inforamtion**

Effects of azo dye on simultaneous biological removal of azo dye and nutrients in wastewater

Aihui Chen^a,b^, Bairen Yang^a^, Yuanqiang Zhou^a^, Yuze Sun^a^, and Cheng Ding^a,^*

^a^School of Environmental Science and Engineering, Yancheng Institute of Technology, Yancheng Jiangsu, 224051, China;

^b^Key Laboratory of tideland ecology and pollution control about environmental protection, Yancheng, Jiangsu, 224051, China

**Table S1.** Characteristics of domestic wastewater used in this study.

| Parameter | Content range |
| --- | --- |
| pH | 6.8-7.2 |
| NH_4_^+^-N (mg/L) | 18.6-24.3 |
| TN (mg/L) | 26.30-32.54 |
| Total phosphorous (mg/L) | 3.57-4.21 |
| COD (mg/L) | 385.4-402.8 |


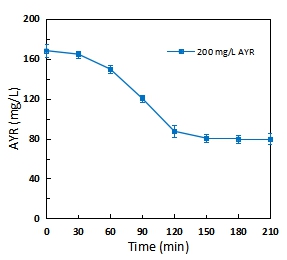


**Figure S1.** Effects of anaerobic time on the AYR decolorization. Error bars represent standard deviations of triplicate tests.


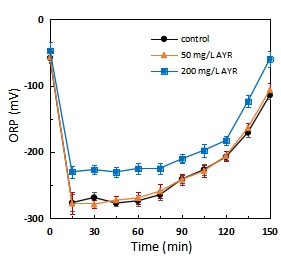


**Figure S2.** Effects of AYR on the variations of ORP during the anaerobic stage in the acclimated activated sludge culture. Error bars represent standard deviations of triplicate tests.

**1. Calculation**

AYR decolorization efficiency was calculated on the concentration difference between influent and effluent, as shown in Eq. (1)

AYR decolorization efficiency = [(AYR_in_-AYR_eff_)/AYR_in_]*100% (1)

Where AYR_in_ and AYR_eff_ are the AYR concentrations in the influent and effluent, separately, mg/L.

COD removal efficiency (mg/L) was defined as the concentration difference between influent and effluent, as shown in Eq. (2):

COD removal efficiency = [(COD_in_ - COD_eff_)/CODin]*100% (2)

Where COD_in_ and COD_eff_ are the COD concentrations in the influent and effluent, separately, mg/L.

Total nitrogen (TN) removal efficiency was calculated based on the concentration difference between influent and effluent, as shown in Eq. (3)

TN removal efficiency = [(TN_in_-TN_eff_)/TN_in_]*100% (3)

Where TN_in_ and TN_eff_ are the TN concentrations in the influent and effluent, separately, mg/L.

**2. AYR concentration measurement**

AYR concentration was quantified by a UV-Vis spectrophotometer (Shimadzu UV-2550, Japan) at a wavelength of 375 nm. First, a standard cure y = 15.625x (*R*^2^ = 0.9962) was established. Then the adsorption value was tested after the wastewater diluted to satisfy the detection limit. Table S2 and S3 show the adsorption values calculated at 375 nm wavelength.

**3. COD concentration measurement**

Table S4 and S4 show the concentration of COD. The COD concentration only refers to the COD from the wastewater, but not including the AYR. So the concentration was calculated based on the Eq. (4)

COD = COD_cr_ - COD_AYR_  (4)

Where COD_AYR_ is the COD value for AYR (1g AYR is equivalent to 1.84 g COD); COD_cr_ (O_2_, mg/L) was tested by Potassium Permanganate Titration and then calculated based on the Eq. (5)

CODcr = [(V_0_-V_1_)×C×8×1000]/V (5)

Where C is the concentration of the ammonium ferrous sulfate standard solution, mol/L; V_0_ and V_1_ are the volumes of ammonium ferrous sulfate standard solution used to back-titrate the control and tested samples, separately, mL; V is the volume of the tested wastewater sample, mL.

**4. NO_2_^-^-N, NO_3_^-^-N, and NH_4_^+^-N concentration measurement**

NH_4_^+^-N, NO_3_^-^-N, and NO_2_^-^-N concentrations were quantified by a UV-Vis spectrophotometer (Shimadzu UV-2550, Japan) at wavelengths of 420, 220 and 275, and 540, separately. First, standard cures were established, y = 266.58x (*R*^2^ = 0.9994) for NH_4_^+^-N, y = 176.81x (*R*^2^ = 0.9973) for NO_3_^-^-N, and y = 15.078x (*R*^2^ = 0.9998) for NO_2_^-^-N. Then the adsorption value was tested after the wastewater diluted to satisfy the detection limit. Table S6 shows the adsorption values for outlet wastewater.

**5. Intracellular ROS production measurement**

Activated sludge was centrifuged at 100 g for 5 min and then was resuspended in 0.1 M phosphate buffer containing 50 μM of dichlorodihydrofluorescein diacetate (H_2_DCF-DA, Molecular Probes, Invitrogen). The mixture was incubated for 30 min at 21 ± 1 ^o^C in dark. After incubation, the phosphate buffer containing H_2_DCF-DA was removed by centrifugation (100 g, 5 min). The pellets were resuspended in wastewater containing the test concentrations (0, 50, and 200 mg/L) of AYR and plated into a 96-well plate. The generated fluorescein DCF was measured after 4.5 h using a microplate reader (BioTek, Winooski, VT) with 485 nm excitation and 520 nm emission filter. The readings are showed in table S7.

**6. LDH activity measurement**

LDH activity was measured with a cytotoxicity detection kit (Roche Applied Science) . At the end of a cycle, the suspension was centrifuged at 12 000 g for 5 min and then the supernatant was seeded on a 96-well plate. After adding 50 μL of substrate mix (Roche Applied Science), the mixture was incubated for 30 min at room temperature in dark. 50 μL of stop solution (Roche Applied Science) was added to each well and the absorbance was recorded at 490 nm using a microplate reader (BioTek). The readings are showed in table S8.

**Raw data**

**Table S2.** The raw data of adsorption values at a wavelength of 375 nm used to calculate the AYR concentration during the 15 days exposure.

|  | | | | | | | | | | | | | | | | | | | | | | | | | | | | | | | | |
| --- | --- | --- | --- | --- | --- | --- | --- | --- | --- | --- | --- | --- | --- | --- | --- | --- | --- | --- | --- | --- | --- | --- | --- | --- | --- | --- | --- | --- | --- | --- | --- | --- |
| Parallel | | | | Sampling time (d) | | | | | | | | | | | | | | | | | | | | | | | | | | | | |
|  |  |  |  | 1 | 2 | | 3 | | 4 | | 5 | 6 | | 7 | | | 8 | | 9 | | 10 | | 11 | | 12 | | 13 | | 14 | | 15 | |
| 1. SBR1 (Inlet AYR concentration of 50 mg/L) | | | | | | | | | | | | | | | | | | | | | | | | | | | | | | | | |
| Inlet ^b^ | |  | | 0.309 | 0.316 | | 0.319 | | 0.318 | | 0.322 | 0.309 | | 0.312 | | | 0.313 | | 0.317 | | 0.311 | | 0.307 | | 0.319 | | 0.316 | | 0.318 | | 0.317 | |
| Outlet ^a^ | | 1 | | 0.303 | 0.555 | | 0.538 | | 0.532 | | 0.377 | 0.524 | | 0.367 | | | 0.382 | | 0.555 | | 0.367 | | 0.368 | | 0.461 | | 0.453 | | 0.414 | | 0.437 | |
|  |  | 2 | | 0.243 | 0.586 | | 0.574 | | 0.479 | | 0.424 | 0.481 | | 0.394 | | | 0.392 | | 0.579 | | 0.375 | | 0.379 | | 0.446 | | 0.475 | | 0.477 | | 0.455 | |
|  |  | 3 | | 0.334 | 0.527 | | 0.498 | | 0.470 | | 0.449 | 0.406 | | 0.433 | | | 0.330 | | 0.543 | | 0.457 | | 0.494 | | 0.476 | | 0.575 | | 0.403 | | 0.413 | |
| 2. SBR2 (Inlet AYR concentration of 200 mg/L) | | | | | | | | | | | | | | | | | | | | | | | | | | | | | | | | |
| Inlet ^c^ | |  | | 0.254 | 0.259 | | 0.258 | | 0.256 | | 0.257 | 0.254 | | 0.254 | | | 0.258 | | 0.258 | | 0.254 | | 0.260 | | 0.256 | | 0.262 | | 0.255 | | 0.255 | |
| Outlet ^b^ | | 1 | | 0.355 | 0.503 | | 0.395 | | 0.359 | | 0.442 | 0.496 | | 0.449 | | | 0.470 | | 0.494 | | 0.518 | | 0.469 | | 0.384 | | 0.423 | | 0.491 | | 0.520 | |
|  |  | 2 | | 0.428 | 0.494 | | 0.390 | | 0.408 | | 0.355 | 0.475 | | 0.369 | | | 0.483 | | 0.519 | | 0.400 | | 0.488 | | 0.416 | | 0.411 | | 0.456 | | 0.461 | |
|  |  | 3 | | 0.372 | 0.391 | | 0.491 | | 0.341 | | 0.365 | 0.407 | | 0.388 | | | 0.400 | | 0.419 | | 0.459 | | 0.376 | | 0.351 | | 0.324 | | 0.371 | | 0.390 | |
| 3. SBR3 (Inlet AYR concentration of 400 mg/L) | | | | | | | | | | | | | | | | | | | | | | | | | | | | | | | | |
| Inlet ^d^ |  | | 0.197 | | | 0.289 | | 0.291 | | 0.287 | | |  | |  |  | |  | |  | |  | |  | |  | |  | |  | |  |
| Outlet ^d^ | 1 | | 0.164 | | | 0.284 | | 0.297 | | 0.288 | | |  | |  |  | |  | |  | |  | |  | |  | |  | |  | |  |
|  | 2 | | 0.197 | | | 0.289 | | 0.287 | | 0.287 | | |  | |  |  | |  | |  | |  | |  | |  | |  | |  | |  |
|  | 3 | | 0.231 | | | 0.296 | | 0.289 | | 0.284 | | |  | |  |  | |  | |  | |  | |  | |  | |  | |  | |  |
| Note: ^a^ The adsorption value was tested in 1^a^, 10 ^b^, 50 ^c^, and 100 ^d^ -fold diluted wastewater and the standard cure is y = 15.625x (*R*^2^ = 0.9962) | | | | | | | | | | | | | | | | | | | | | | | | | | | | | | | | |

**Table S3.** The raw data of adsorption values at a wavelength of 375 nm used to calculate the variation of AYR during one cycle.

|  | | Sampling time (min) | | | | | | | | | | | | | | |
| --- | --- | --- | --- | --- | --- | --- | --- | --- | --- | --- | --- | --- | --- | --- | --- | --- |
|  |  | 0 | 30 | 60 | 90 | 120 | 150 | 180 | 210 | 240 | 270 | 300 | 330 | 360 | 390 |  |
| 50 mg/L^a^ | 1 | 0.199 | 0.278 | 0.193 | 0.157 | 0.087 | 0.054 | 0.074 | 0.060 | 0.054 | 0.051 | 0.056 | 0.050 | 0.055 | 0.053 |  |
|  | 2 | 0.254 | 0.233 | 0.191 | 0.053 | 0.043 | 0.029 | 0.028 | 0.033 | 0.036 | 0.036 | 0.034 | 0.042 | 0.035 | 0.035 |  |
|  | 3 | 0.298 | 0.189 | 0.129 | 0.122 | 0.057 | 0.067 | 0.041 | 0.045 | 0.041 | 0.039 | 0.036 | 0.032 | 0.034 | 0.033 |  |
| 200 mg/L^b^ | 1 | 0.227 | 0.216 | 0.197 | 0.161 | 0.124 | 0.110 | 0.102 | 0.098 | 0.103 | 0.100 | 0.103 | 0.101 | 0.100 | 0.097 |  |
|  | 2 | 0.207 | 0.213 | 0.194 | 0.150 | 0.104 | 0.105 | 0.106 | 0.088 | 0.102 | 0.100 | 0.100 | 0.088 | 0.098 | 0.101 |  |
|  | 3 | 0.212 | 0.204 | 0.184 | 0.151 | 0.108 | 0.095 | 0.092 | 0.109 | 0.087 | 0.091 | 0.086 | 0.099 | 0.090 | 0.090 |  |
| Note: The adsorption value was tested in 10 ^a^ and 50 ^b^ fold diluted wastewater and the standard cure is y = 15.625x (*R*^2^ = 0.9962). | | | | | | | | | | | | | | | | |

**Table S4.** The raw data of COD concentrations (mg/L) during the 15 days exposure.

| Parallel | | Sampling time (d) | | | | | | | | | | | | | | | |
| --- | --- | --- | --- | --- | --- | --- | --- | --- | --- | --- | --- | --- | --- | --- | --- | --- | --- |
|  |  | 0 | 1 | 2 | 3 | 4 | 5 | 6 | 7 | 8 | 9 | 10 | 11 | 12 | 13 | 14 | 15 |
| 1. Control SBR | | | | | | | | | | | | | | | | | |
| Inlet |  | 393.58 | 390.98 | 402.38 | 394.78 | 393.24 | 394.31 | 384.98 | 399.48 | 390.48 | 392.08 | 395.58 | 401.28 | 404.48 | 386.38 | 391.28 | 393.28 |
| Outlet | 1 | 27.69 | 57.32 | 41.28 | 52.50 | 46.31 | 67.74 | 64.50 | 37.33 | 54.38 | 41.54 | 62.37 | 30.44 | 41.05 | 58.75 | 54.62 | 33.95 |
|  | 2 | 34.23 | 47.80 | 38.74 | 37.35 | 34.58 | 68.37 | 62.18 | 35.12 | 52.16 | 52.30 | 50.96 | 35.74 | 29.37 | 43.38 | 51.39 | 46.80 |
|  | 3 | 43.12 | 46.35 | 29.42 | 47.61 | 42.57 | 62.15 | 46.89 | 47.67 | 46.69 | 53.55 | 54.38 | 47.33 | 31.15 | 47.83 | 44.36 | 43.67 |
| 2. SBR1 (inlet AYR concentration of 50 mg/L) | | | | | | | | | | | | | | | | | |
| Inlet |  | 402.80 | 393.11 | 396.91 | 398.41 | 400.71 | 389.51 | 393.31 | 398.51 | 396.51 | 399.62 | 401.21 | 400.51 | 400.51 | 396.81 | 396.01 | 398.71 |
| Outlet | 1 | 37.84 | 51.45 | 45.65 | 43.34 | 36.30 | 44.92 | 50.85 | 43.32 | 38.95 | 47.77 | 30.79 | 35.30 | 45.52 | 38.39 | 49.59 | 43.93 |
|  | 2 | 34.34 | 43.19 | 40.82 | 36.39 | 32.17 | 55.37 | 44.85 | 43.32 | 45.25 | 33.61 | 26.52 | 39.09 | 32.86 | 36.65 | 42.12 | 35.51 |
|  | 3 | 27.05 | 42.53 | 36.57 | 37.73 | 40.51 | 50.25 | 40.70 | 56.48 | 40.30 | 33.47 | 23.73 | 35.27 | 31.27 | 48.34 | 34.65 | 36.89 |
| 3. SBR2 (Inlet AYR concentration of 200 mg/L) | | | | | | | | | | | | | | | | | |
| Inlet |  | 397.42 | 401.29 | 398.47 | 396.40 | 396.34 | 397.50 | 394.90 | 387.81 | 396.81 | 389.50 | 396.70 | 399.37 | 397.22 | 400.30 | 393.6 | 396.70 |
| Outlet | 1 | 40.17 | 73.35 | 139.93 | 129.56 | 100.44 | 65.49 | 55.31 | 85.29 | 77.54 | 76.44 | 61.62 | 63.09 | 65.33 | 54.51 | 68.73 | 80.70 |
|  | 2 | 39.45 | 104.39 | 125.10 | 112.15 | 92.34 | 75.54 | 53.00 | 74.51 | 60.38 | 58.37 | 65.48 | 67.37 | 69.00 | 67.28 | 56.14 | 58.57 |
|  | 3 | 34.71 | 115.52 | 116.55 | 107.65 | 87.50 | 67.39 | 72.65 | 80.56 | 71.28 | 64.91 | 54.53 | 52.18 | 56.78 | 60.80 | 60.58 | 70.15 |

**Table S5.** The raw data of COD concentration (mg/L) during one cycle.

| Parallel | | Sampling time (min) | | | | | | | | | | | | | |
| --- | --- | --- | --- | --- | --- | --- | --- | --- | --- | --- | --- | --- | --- | --- | --- |
|  |  | 0 | 30 | 60 | 90 | 120 | 150 | 180 | 210 | 240 | 270 | 300 | 330 | 360 | 390 |
| control | 1 | 299.47 | 254.47 | 197.65 | 172.25 | 144.32 | 143.55 | 116.67 | 72.43 | 60.8 | 47.51 | 40.41 | 34.14 | 33.42 | 33.21 |
|  | 2 | 296.43 | 241.35 | 214.78 | 162.83 | 147.39 | 138.74 | 94.30 | 65.18 | 51.12 | 38.51 | 34.28 | 31.16 | 29.95 | 30.23 |
|  | 3 | 318.54 | 240.06 | 197.55 | 153.65 | 151.28 | 145.50 | 106.25 | 64.42 | 47.75 | 37.73 | 34.78 | 32.00 | 30.84 | 30.81 |
| 50 mg/L^a^ | 1 | 322.11 | 258.38 | 226.48 | 185.65 | 160.49 | 157.45 | 126.44 | 83.47 | 59.98 | 47.27 | 43.05 | 38.82 | 35.84 | 35.47 |
|  | 2 | 310.17 | 255.59 | 224.74 | 168.92 | 161.55 | 154.4 | 117.49 | 69.52 | 51.85 | 42.68 | 33.62 | 33.14 | 33.15 | 32.78 |
|  | 3 | 332.34 | 240.66 | 210.8 | 172.31 | 165.97 | 160.32 | 115.28 | 73.14 | 53.67 | 41.25 | 38.25 | 34.90 | 34.27 | 34.01 |
| 200 mg/L^b^ | 1 | 369.50 | 314.28 | 281.29 | 248.6 | 220.69 | 211.15 | 181.15 | 145.27 | 121.39 | 95.74 | 81.55 | 75.69 | 72.47 | 68.95 |
|  | 2 | 351.17 | 301.57 | 263.90 | 239.83 | 216.82 | 206.58 | 159.54 | 131.55 | 109.86 | 88.88 | 74.38 | 70.57 | 66.94 | 64.61 |
|  | 3 | 372.34 | 310.64 | 266.34 | 236.35 | 206.14 | 201.96 | 163.39 | 141.00 | 103.75 | 83.57 | 73.45 | 68.84 | 64.53 | 64.25 |

**Table S6.** The raw data of adsorption values used to calculate the concentrations of NO_2_^-^-N, NO_3_^-^-N, and NH_4_^+^-N.

|  | | Sampling time (d) | | | | | | | | | | | | | | |
| --- | --- | --- | --- | --- | --- | --- | --- | --- | --- | --- | --- | --- | --- | --- | --- | --- |
|  |  | 1 | 2 | 3 | 4 | 5 | 6 | 7 | 8 | 9 | 10 | 11 | 12 | 13 | 14 | 15 |
| 1. control SBR | | | | | | | | | | | | | | | | |
| A420^a^ | 1 | 0.042 | 0.07 | 0.088 | 0.05 | 0.042 | 0.038 | 0.034 | 0.03 | 0.038 | 0.03 | 0.028 | 0.084 | 0.05 | 0.042 | 0.034 |
|  | 2 | 0.056 | 0.046 | 0.090 | 0.036 | 0.030 | 0.030 | 0.052 | 0.008 | 0.076 | 0.018 | 0.049 | 0.074 | 0.036 | 0.048 | 0.018 |
|  | 3 | 0.052 | 0.082 | 0.058 | 0.042 | 0.024 | 0.016 | 0.034 | 0.012 | 0.058 | 0.014 | 0.062 | 0.070 | 0.036 | 0.050 | 0.016 |
| A220-2*A275^c^ | 1 | 0.130 | 0.871 | 0.235 | 0.850 | 0.080 | 1.430 | 0.325 | 0.448 | 0.888 | 0.338 | 0.740 | 0.155 | 0.840 | 0.830 | 0.323 |
|  | 2 | 0.133 | 0.879 | 0.223 | 0.893 | 0.103 | 0.865 | 0.318 | 0.423 | 0.845 | 0.318 | 0.798 | 0.128 | 0.833 | 0.868 | 0.313 |
|  | 3 | 0.143 | 0.880 | 0.283 | 0.758 | 0.105 | 1.215 | 0.290 | 0.428 | 0.793 | 0.298 | 0.763 | 0.130 | 0.873 | 0.830 | 0.320 |
| A540^f^ | 1 | 0.128 | 0.312 | 0.498 | 0.215 | 0.375 | 0.271 | 0.25 | 0.265 | 0.582 | 0.415 | 0.029 | 0.115 | 0.145 | 0.057 | 0.142 |
|  | 2 | 0.139 | 0.279 | 0.506 | 0.23 | 0.356 | 0.243 | 0.243 | 0.312 | 0.618 | 0.381 | 0.032 | 0.100 | 0.124 | 0.068 | 0.127 |
|  | 3 | 0.132 | 0.303 | 0.487 | 0.204 | 0.364 | 0.281 | 0.204 | 0.318 | 0.492 | 0.397 | 0.038 | 0.082 | 0.131 | 0.073 | 0.129 |
| 2. SBR1 (Inlet AYR concentration of 50 mg/L) | | | | | | | | | | | | | | | | |
| A420^a^ | 1 | 0.095 | 0.075 | 0.069 | 0.075 | 0.085 | 0.102 | 0.064 | 0.087 | 0.095 | 0.075 | 0.080 | 0.077 | 0.079 | 0.070 | 0.085 |
|  | 2 | 0.083 | 0.078 | 0.085 | 0.106 | 0.100 | 0.083 | 0.066 | 0.069 | 0.113 | 0.098 | 0.075 | 0.105 | 0.083 | 0.071 | 0.059 |
|  | 3 | 0.071 | 0.065 | 0.084 | 0.082 | 0.104 | 0.068 | 0.057 | 0.096 | 0.079 | 0.079 | 0.065 | 0.055 | 0.075 | 0.073 | 0.054 |
| A220-2*A275^d^ | 1 | 0.207 | 0.262 | 0.336 | 0.314 | 0.286 | 0.169 | 0.241 | 0.265 | 0.272 | 0.261 | 0.296 | 0.336 | 0.348 | 0.315 | 0.241 |
|  | 2 | 0.225 | 0.310 | 0.336 | 0.358 | 0.195 | 0.166 | 0.222 | 0.229 | 0.183 | 0.316 | 0.350 | 0.255 | 0.315 | 0.202 | 0.352 |
|  | 3 | 0.237 | 0.310 | 0.302 | 0.319 | 0.142 | 0.221 | 0.248 | 0.195 | 0.215 | 0.311 | 0.275 | 0.252 | 0.289 | 0.254 | 0.294 |
| A540^g^ |  | 0.147 | 0.320 | 0.325 | 0.315 | 0.312 | 0.215 | 0.253 | 0.200 | 0.261 | 0.285 | 0.193 | 0.220 | 0.265 | 0.207 | 0.179 |
|  |  | 0.139 | 0.339 | 0.284 | 0.330 | 0.299 | 0.183 | 0.203 | 0.118 | 0.328 | 0.182 | 0.201 | 0.244 | 0.221 | 0.128 | 0.138 |
|  |  | 0.151 | 0.355 | 0.312 | 0.276 | 0.260 | 0.189 | 0.225 | 0.157 | 0.342 | 0.205 | 0.158 | 0.247 | 0.195 | 0.123 | 0.167 |
| 3. SBR2 (Inlet AYR concentration of 200 mg/L) | | | | | | | | | | | | | | | | |
| A420^b^ | 1 | 0.054 | 0.287 | 0.429 | 0.460 | 0.301 | 0.197 | 0.264 | 0.356 | 0.293 | 0.158 | 0.134 | 0.179 | 0.175 | 0.277 | 0.245 |
|  | 2 | 0.095 | 0.374 | 0.504 | 0.389 | 0.238 | 0.152 | 0.286 | 0.355 | 0.305 | 0.114 | 0.255 | 0.134 | 0.383 | 0.310 | 0.251 |
|  | 3 | 0.212 | 0.395 | 0.477 | 0.330 | 0.194 | 0.152 | 0.275 | 0.356 | 0.359 | 0.115 | 0.185 | 0.165 | 0.390 | 0.245 | 0.217 |
| A220-2*A275^e^ | 1 | 0.078 | 0.314 | 0.329 | 0.247 | 0.280 | 0.277 | 0.244 | 0.254 | 0.246 | 0.197 | 0.235 | 0.231 | 0.218 | 0.243 | 0.239 |
|  | 2 | 0.125 | 0.317 | 0.295 | 0.295 | 0.257 | 0.277 | 0.239 | 0.265 | 0.263 | 0.221 | 0.249 | 0.187 | 0.285 | 0.189 | 0.288 |
|  | 3 | 0.187 | 0.345 | 0.278 | 0.271 | 0.292 | 0.232 | 0.255 | 0.217 | 0.251 | 0.198 | 0.230 | 0.212 | 0.274 | 0.211 | 0.264 |
| A540^h^ | 1 | 0.095 | 0.314 | 0.374 | 0.300 | 0.248 | 0.293 | 0.237 | 0.278 | 0.207 | 0.385 | 0.183 | 0.260 | 0.265 | 0.347 | 0.248 |
|  | 2 | 0.102 | 0.358 | 0.381 | 0.310 | 0.297 | 0.213 | 0.318 | 0.332 | 0.158 | 0.379 | 0.192 | 0.247 | 0.351 | 0.328 | 0.198 |
|  | 3 | 0.151 | 0.365 | 0.347 | 0.276 | 0.301 | 0.250 | 0.315 | 0.353 | 0.182 | 0.300 | 0.159 | 0.234 | 0.351 | 0.300 | 0.173 |
| Note: The NH_4_^+^-N concentration was calculated by the standard cure ^a^ y =5.3316x and ^b^ y =26.658x (*R*^2^ = 0.9994). The NO_3_^-^-N concentration was calculated by the standard cure ^c^ y = 7.0724x, ^d^ y = 17.681x, and ^e^ y = 35.362x (*R*^2^ = 0.9973). The NO_2_^-^-N concentration was calculated by the standard cure ^f^ y = 0.30156x, ^g^ y = 0.60312x, and ^h^ y = 1.5078x (*R*^2^ = 0.9998). | | | | | | | | | | | | | | | | |

**Table S7.** The values of the generated fluorescein DCF used to calculate the ROS production.

|  | Parallel | | |
| --- | --- | --- | --- |
|  | 1 | 2 | 3 |
| control | 0.158 | 0.163 | 0.142 |
| 50 mg/L AYR | 0.170 | 0.152 | 0.166 |
| 200 mg /L AYR | 0.217 | 0.203 | 0.179 |

**Table S8.** The values used to calculate the LDH activity.

|  | Parallel | | |
| --- | --- | --- | --- |
|  | 1 | 2 | 3 |
| control | 0.098 | 0.094 | 0.104 |
| 50 mg/L AYR | 0.098 | 0.105 | 0.089 |
| 200 mg /L AYR | 0.108 | 0.097 | 0.104 |
